# Association between amino acids and recent osteoporotic fracture: a matched incident case-control study

**DOI:** 10.3389/fnut.2024.1360959

**Published:** 2024-03-19

**Authors:** Bing Liang, Xinyan Shi, Xinwei Wang, Chao Ma, William D. Leslie, Lisa M. Lix, Xianbao Shi, Bo Kan, Shuman Yang

**Affiliations:** ^1^Department of Endocrinology, The First Affiliated Hospital of Jinzhou Medical University, Jinzhou, Liaoning, China; ^2^Center for Mitochondrial Biology and Medicine, The Key Laboratory of Biomedical Information Engineering of Ministry of Education, School of Life Science and Technology, Xi’an Jiao tong University, Xi’an, China; ^3^Department of Epidemiology and Biostatistics, School of Public Health, Jilin University, Changchun, Jilin, China; ^4^Department of Internal Medicine, University of Manitoba, Winnipeg, MB, Canada; ^5^Department of Community Health Sciences, University of Manitoba, Winnipeg, MB, Canada; ^6^Department of Pharmacy, The First Affiliated Hospital of Jinzhou Medical University, Jinzhou, Liaoning, China; ^7^Department of Clinical Laboratory, The Second Hospital of Jilin University, Changchun, Jilin, China

**Keywords:** fracture, amino acids, osteoporosis, metabolomics, bone health

## Abstract

**Context:**

Osteoporotic fracture is a major public health issue globally. Human research on the association between amino acids (AAs) and fracture is still lacking.

**Objective:**

To examine the association between AAs and recent osteoporotic fractures.

**Methods:**

This age and sex matched incident case-control study identified 44 recent x-ray confirmed fracture cases in the Second Hospital of Jilin University and 88 community-based healthy controls aged 50+ years. Plasma AAs were measured by high performance liquid chromatography coupled with mass spectrometry. After adjusting for covariates (i.e., body mass index, milk intake >1 time/week, falls and physical activity), we conducted conditional logistical regression models to test the association between AAs and fracture.

**Results:**

Among cases there were 23 (52.3%) hip fractures and 21 (47.7%) non-hip fractures. Total, essential, and non-essential AAs were significantly lower in cases than in controls. In the multivariable conditional logistic regression models, after adjusting for covariates, each standard deviation increase in the total (odds ratio [OR]: 0.304; 95% confidence interval [CI]: 0.117–0.794), essential (OR: 0.408; 95% CI: 0.181–0.923) and non-essential AAs (OR: 0.290; 95%CI: 0.107–0.782) was negatively associated with recent fracture. These inverse associations were mainly found for hip fracture, rather than non-hip fractures. Among these AAs, lysine, alanine, arginine, glutamine, histidine and piperamide showed the significantly negative associations with fracture.

**Conclusion:**

There was a negative relationship between AAs and recent osteoporotic fracture; such relationship appeared to be more obvious for hip fracture.

## 1 Introduction

Osteoporotic fracture is a major public health problem in China. The prevalence of vertebral fractures in men and women aged 40 years or above was 10.5 and 9.7%, respectively (1). Complications such as constipation, stroke, pneumonia and arrhythmias increase after an osteoporotic fracture (2). The total 5-year mortality rates in fracture patients are 39% for women and 51% for men (3). In western China, the mean total costs for hip, vertebral and wrist fractures of the first year were estimated at RMB 57,585 per patient (approximately 9,140 US dollars) (4).

Fracture is a major clinical consequence of osteoporosis, a skeletal disease characterized by decreased bone mass and deteriorated bone microstructure (5). In both animal and human studies, AAs are linked with bone health. After 9 weeks of feeding arginine supplements, the femur bone mineral density (BMD) in experimental rats was significantly higher than in a control group that did not receive the supplements (6). In humans, higher levels of valine, leucine, isoleucine and tryptophan were associated with decreased hip BMD decline and higher BMD (7, 8). Valine, alanine, histidine and tryptophan were inversely associated with osteoporosis risk (9, 10).

Although there is evidence suggesting a link between AAs and BMD, few studies have examined the relationship between AAs and osteoporotic fracture. Lower plasma levels of ornithine, taurine, and aspartic acid were found in fracture patients when compared with controls (11). A previous case-control study also showed that the majority of essential and non-essential amino acids in fracture patients were significantly lower compared with healthy controls (12). Further, compared with healed-fracture patients, hypertrophic and atrophic nonunion patients had significantly lower levels of arginine and citrulline, respectively (13).

Thus, the aim of the present study was to examine the association between AAs and recent osteoporotic fractures. Previous research has shown the beneficial role of AAs for maintaining muscle and kidney health (14, 15). This research may help to extend our understanding about the relationship between AAs and bone health including bone healing.

## 2 Materials and methods

### 2.1 Study setting and subjects

Similar to our previous study (16), incident cases were confirmed to be new fractures in the Second Hospital of Jilin University in 2020. Using the survivor sampling methods, we selected controls from a community-based generally healthy population who had no history of fracture in Changchun, Jilin in 2020. Individuals who were 50 years or older and had never used osteoporosis-related medications were enrolled. We excluded controls with secondary osteoporosis. Cases with pathological fractures or incomplete fracture information were excluded. Cases were matched with controls by age (±4 years) and sex in a ratio of 1:2. Based on a pilot study of 4 fracture cases and 8 controls, and their corresponding total AA levels (1226.68 ± 301.00 μmol/L in cases and 1446.97 ± 94.57 μmol/L in controls), to achieve study power > 0.80 with a = 0.05, we estimated minimum sample sizes for cases and controls of 11 and 22, respectively. Written informed consent was obtained from all individual participants. The study protocol was approved by the institutional review boards (IRBs) of the School of Public Health, Jilin University (Project #: 2022-02-02).

### 2.2 Blood collection and measurement of amino acids

The blood collection method was described in a previous study (16). For cases, we collected blood samples within 2 days after hospitalization for fracture, but before any treatment (i.e., fracture fixation, hip replacement and medications). Blood samples for controls were collected at the time of interview. The blood samples were processed and refrigerated at −80°C until tested.

Circular pieces of plasma filter paper were created with a diameter of 3 mm. The metabolites were extracted with ethanol and the supernatant was extracted after centrifugation. After filtration, the supernatant was transferred to a 96-well plate. AA metabolite standards (Cambridge Isotope Laboratory, Tewksbury, MA, United States) and AA quality control solution were likewise transferred to the 96-well plate. The 96-well plate was first dried with nitrogen, then cultured with a 1-butanol acetyl chloride mixture and then dried again with nitrogen. The test sample was mixed with mobile phase solution (80% acetonitrile aqueous solution) and detected by high performance liquid chromatography coupled with mass spectrometry.

All AAs were classified as essential or non-essential. Essential AAs included leucine, lysine, methionine, phenylalanine, threonine, tryptophan and valine; non-essential AAs were alanine, asparagine, aspartic acid, arginine, citrulline, cysteine, glutamine, glutamic acid, glycine, homocysteine, histidine, ornithine, piperamide, proline, serine and tyrosine. Aromatic AAs such as phenylalanine, tyrosine, tryptophan and branched-chain AAs such as leucine and valine were also considered. Consistent with a previous study (12), essential, non-essential, aromatic, branched-chain, and total AAs were summed together for testing their overall effects. We also calculated AA ratios such as glycine to alanine, methionine to phenylalanine and valine to phenylalanine to determine the effects of specific AA catabolism.

### 2.3 Scertainment of covariates

Consistent with a previous study (16), we included the following covariates in this study: demographics (sex and age), lifestyle factors (e.g., physical activity, smoking status, milk intake >1 time/week and calcium supplement), postmenopausal status in females, disease history (e.g., coronary heart disease, type 2 diabetes, stroke), height loss of more than 3 cm after age 40 years, falls from standing height or less within the last 12 months, family history of osteoporosis and fractures, and body mass index (BMI). These factors were included because they were major risk factors for fractures (17, 18). Except for the measured body weight and height in controls, body weight and height in cases and all the other data were collected via face-to-face methods using a structured questionnaire. BMI was computed as weight divided by height^2^ (kg/m^2^).

### 2.4 Statistical analysis

We descriptively analyzed the baseline characteristics and AA levels by fracture status using frequencies, percentages, means and standard deviations (SD). We conducted multivariable conditional logistical regression models, which account for matching pairs of cases and controls, to test the association between AAs and fracture. AA values that followed a normal distribution, as assessed by skewness and kurtosis, were scaled per 1-SD increase. For AA values that were not normally distributed, values were expressed per 1-SD increase on the logarithmic scale. We adjusted for BMI, physical activity, milk intake >1 time/week and falls in the models, because they showed bivariate associations with fracture at alpha = 0.1. Model fit was assessed by examining *R*^2^ of AAs associated with fracture (0.392). Multiple testing was addressed using false discovery rate (FDR) analysis, which means the proportion of false discoveries (19). Since homocysteine is suggested to have negative impact on fracture risk (20), we estimated the association between AAs and fracture after excluding homocysteine from the analysis. Subgroup analyses by hip and non-hip fracture were also performed; we used all controls to increase the study power and adjusted for age, sex, body mass index, physical activity, smoking, milk intake >1 time/week, calcium supplement, history of coronary heart disease, type 2 diabetes and stroke, height loss >3 cm, falls, family history of osteoporosis and fractures in the unconditional logistic regression models. Lastly, we tested the association of aromatic and branched-chain AAs and AA ratios with fracture risk in the conditional logistic regression models. All conditional regression models were further adjusted for age and sex to avoid potential collider bias. Descriptive and logistic regression analyses were performed using the SPSS software (version 24.0; SPSS, Chicago, IL). We used the “MatchIt” and “fdrtool” packages in the R language (R 4.1.2) to conduct case-control matching and FDR analyses, respectively.

## 3 Results

### 3.1 Descriptive data

In this matched case-control study, we included 88 non-fracture controls and 44 fracture cases, which involved 23 (52.3%) hip fracture patients and 21 (47.7%) non-hip fracture patients (Table 1). Most fractures were due to falls (38.6%) or low-trauma sports injury (38.6%). As compared with controls, cases had significantly lower physical activity and milk intake >1 time/week, and a higher prevalence of falls; other characteristics including age, sex, BMI, smoking, calcium supplement, history of coronary heart disease, type 2 diabetes and stroke, height loss >3 cm, family history of osteoporosis and fractures, were not significantly different between cases and controls. All descriptive data by fracture status have been published (16).

**Table 1:** Amino acid levels by fracture status.

Amino acid (μmol/L; abbreviation)	Fracture cases *N* = 44	Controls *N* = 88	*p*	False discovery rate
**Essential amino acid**				
Lysine (Lys)	105.0 ± 27.8	123.2 ± 23.0	**<0.001**	0.003
Methionine (Met)	8.6 ± 1.9	8.8 ± 1.6	0.576	0.611
Tryptophan (Trp)	32.3 ± 7.8	36.4 ± 7.2	**0.004**	0.009
Threonine (Thr)	24.2 ± 7.3	27.0 ± 6.0	**0.022**	0.039
Leucine (Leu)*	60.1 (48.6, 68.0)	55.2 (49.3, 64.9)	0.224	0.299
Phenylalanine (Phe)*	28.4 (25.0, 32.9)	24.9 (22.6, 28.2)	**0.001**	0.003
Valine (Val)*	85.6 (75.1, 98.1)	90.3 (82.2, 97.1)	0.277	0.330
Total essential amino acid (EAA)*	348.3 (295.0,396.4)	366.2 (339.8, 394.4)	**0.018**	0.038
**Non-essential amino acid**				
Alanine (Ala)	106.5 ± 38.2	151.3 ± 35.8	**<0.001**	0.003
Arginine (Arg)	32.2 ± 10.9	42.9 ± 9.7	**<0.001**	0.003
Cysteine (Cys)	2.0 ± 0.9	2.0 ± 0.7	0.610	0.611
Glutamine (Gln)	5.2 ± 1.4	6.1 ± 1.2	**<0.001**	0.003
Homocysteine (Hcy)	10.9 ± 0.8	10.7 ± 0.7	0.169	0.264
Histidine (His)	33.7 ± 8.3	41.8 ± 7.1	**<0.001**	0.003
Ornithine (Orn)	25.9 ± 8.1	29.6 ± 8.5	**0.020**	0.039
Piperamide (Pip)	222.8 ± 59.6	260.7 ± 48.5	**<0.001**	0.003
Proline (Pro)	162.1 ± 64.8	175.3 ± 53.3	0.215	0.299
Tyrosine (Tyr)	29.8 ± 8.2	31.4 ± 6.7	0.250	0.313
Asparagine (Asn)*	40.8 (37.3,47.2)	39.5(36.4, 44.2)	0.227	0.299
Aspartic acid (Asp)*	6.8 (5.7, 7.7)	6.4 (5.6, 7.3)	0.611	0.611
Citrulline (Cit)*	12.8 (9.3, 16.7)	16.4 (13.9,19.6)	**<0.001**	0.003
Glutamic acid (Glu)	57.9 (48.8, 68.5)	61.8 (54.3, 69.4)	0.091	0.152
Glycine (Gly)*	61.2 (53.0, 76.6)	71.2 (63.1, 89.5)	**<0.001**	0.003
Serine (Ser)*	12.6 (11.5, 15.4)	13.4 (11.9, 15.1)	0.431	0.490
Total non-essential amino acid (NEAA)	835.7 ± 170.5	974.5 ± 136.3	**<0.001**	0.003
Total amino acid (TAA)	1180.6 ± 219.1	1344.5 ± 176.8	**<0.001**	0.003

Amino acid levels following a normal distribution are presented as mean ± standard deviation (SD); *non-normally distributed data are presented as median (interquartile range). Bold-faced values indicate statistically significant at alpha = 0.05.

### 3.2 Association between AAs and fracture

**Figure 1:**
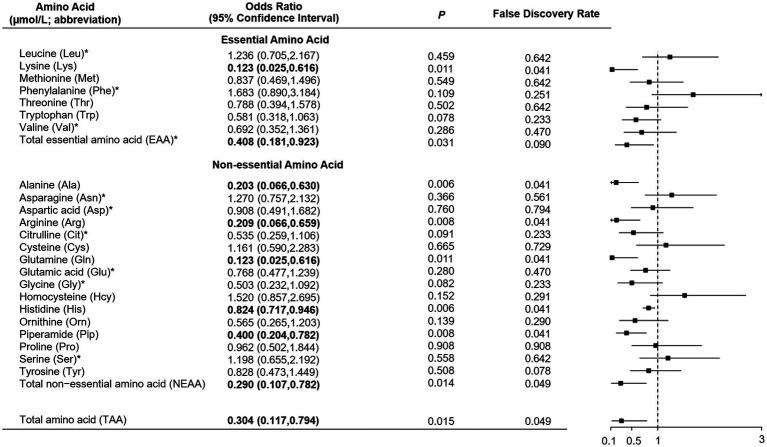
Multivariable conditional logistic regression analysis of the association between amino acid levels and fracture. *Odds ratio presented per 1-SD increase on the logarithmic scale; all the other odds ratios are presented per 1-SD increase on the linear scale. Odds ratios were adjusted for body mass index, physical activity, milk intake >1 time/week and falls.

**Figure 2:**
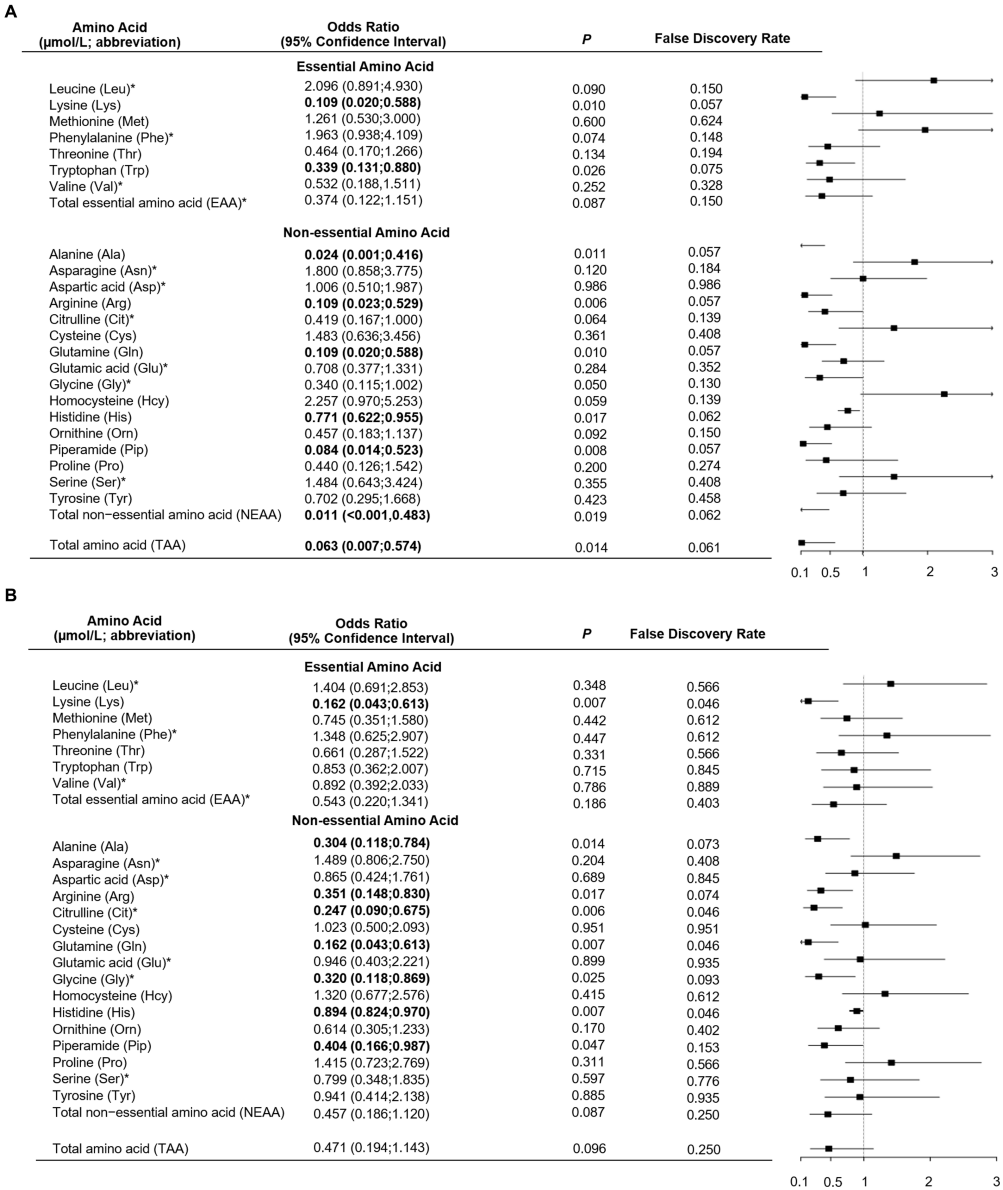
Multivariable unconditional logistic regression analysis of the association of amino acid levels with hip fracture **(A)** and non-hip fracture **(B)**. *Odds ratio presented per 1-SD increase on the logarithmic scale; all the other odds ratios are presented per 1-SD increase on the linear scale. Odds ratios were adjusted for age, sex, body mass index, physical activity, smoking, milk intake >1 time/week, calcium supplement, history of coronary heart disease, type 2 diabetes and stroke, height loss >3 cm, falls, family history of osteoporosis and fractures.

**Figure 3:**
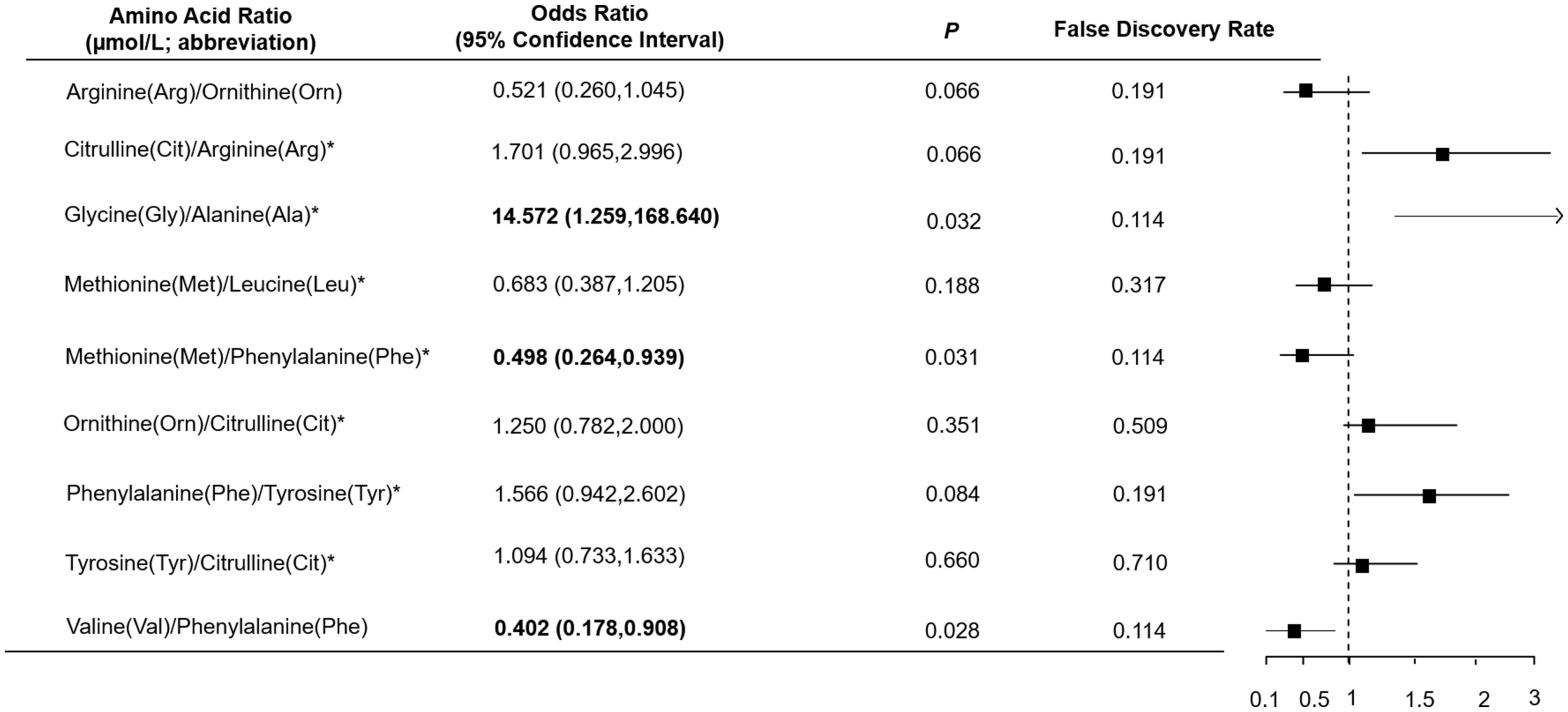
Multivariable logistic regression analysis of the association between amino acid ratio and fracture. *Odds ratio presented per 1-SD increase on the logarithmic scale; all the other odds ratios are presented per 1-SD increase on the linear scale. Odds ratios were adjusted for body mass index, physical activity, milk intake >1 time/week and falls.

As shown in Table 1, total, essential and non-essential AAs were significantly lower in cases than controls. In the multivariable conditional logistic regression analysis, after adjusting for BMI, physical activity, milk intake >1 time/week and falls, total (odds ratio [OR]: 0.304; 95% confidence interval [CI]: 0.117–0.794; Figure 1), essential (OR: 0.408; 95% CI: 0.181–0.923) and non-essential AAs (OR: 0.290; 95% CI: 0.107–0.782) were negatively associated with fracture. Similar results were noted when we further adjusted for age and sex (Supplementary Figure S1). The FDRs for total, essential and non-essential AAs were 0.049, 0.09, and 0.049, respectively. After excluding homocysteine from analysis, the non-essential (OR: 0.289; 95%CI: 0.107–0.780) and total AAs (OR: 0.304; 95%CI: 0.117–0.793) also showed negative association with fracture. For the essential AAs, lysine showed the strongest association with fractures. Among the non-essential AAs, alanine, arginine, glutamine, histidine and piperamide levels were negatively associated with fracture. The negative relationship between AAs and fractures was mainly observed for hip fracture (Figure 2). Although aromatic and branched-chain AAs showed negative trends with fracture risk, ORs did not reach statistical significance (Supplementary Figure S2). The ratio of glycine to alanine was positively associated with fracture (Figure 3). In contrast, the ratios of methionine to phenylalanine and valine to phenylalanine were negatively associated with fracture. Again, these AA ratios had identical associations with fractures after further adjusting for age and sex (Supplementary Figure S3).

## 4 Discussion

In this 1:2 individually matched case-control study, we found that higher levels of total, essential and non-essential AAs were negatively associated with osteoporotic fracture. The association was mainly found for hip fracture, rather than non-hip fracture. Among these AAs, lysine, alanine, arginine, glutamine, histidine and piperamide showed the strongest negative associations with fracture. The ratio of glycine to alanine was positively associated with fracture, while ratios of methionine to phenylalanine and valine to phenylalanine were negatively associated with fracture.

There is scant research to date investigating the association between AAs and osteoporotic fracture. Our study findings partially agree with a previous study, which found that levels of arginine and glutamine in men with normal BMD compared to those with osteopenia/osteoporosis were higher (21). Lysine may promote bone health through its ability to decrease urinary calcium excretion and enhance calcium absorption (22). Valine, tyrosine, and tryptophan levels have positive associations with BMD (10, 23). Consistent with this, we observed a non-significant inverse relationship between these AAs and osteoporotic fracture. Higher serum homocysteine was suggested to be a risk factor for fracture in postmenopausal women (20); a similar trend was observed in our study.

Our findings that higher valine to phenylalanine and lower glycine to alanine ratios were inversely associated with fracture are supported by prior research (9) which found that higher plasma levels of valine and alanine were negatively associated with osteoporosis in women. Similarly, in the female population, higher glycine levels were associated with lower BMD (24). Methionine was demonstrated to enhance osteoblast proliferation, activation, differentiation and BMD (25, 26), which is in line with our study finding that the ratio of methionine to phenylalanine is negatively associated with fracture.

Fracture remains a major public health issue. The negative association between AAs and fractures could have clinical implications for fracture healing. Significant catabolic response after fracture surgery has been found in a study, which showed that the levels of total AAs were lower in new fracture patients as compared with controls (12). Similarly, compared with healthy population and healed-fracture patients, the fracture and hypertrophic/atrophic nonunion patients exhibited lower levels of certain AAs (11, 13). A two-month human study showed that essential amino acid supplements could significantly increase the concentration of serum album (27). Among elderly fracture patients, higher albumin levels predict greater discharge Functional Independence Measurement scores (28), which is a functional status instrument for rehabilitation inpatients (29). Lower levels of serum albumin were significantly associated with greater length of stay and in-hospital mortality in institutionalized patients with hip fracture (30). A randomized controlled trial demonstrated that after two-months of essential amino acid supplements, fracture patients with sarcopenia showed significant improvements in appendicular muscle strength and physical performance (31). During 20 weeks of feeding foods rich in arginine and lysine, rats in the test group exhibited better fracture healing as compared with the control group (32). Several mechanisms may account for this beneficial effect. Nitric oxide is derived from l-arginine (33). Supplements of arginine increase the synthesis of nitric oxide, which could increase vascularity and angiogenesis (32). Lastly, l-lysine could stimulate intestinal absorption and renal conservation of calcium, which is significantly associated with increased BMD (34, 35).

Some study limitations are acknowledged. Although we found fracture patients had lower AAs than controls, the specific impact of fracture and its related factors (i.e., hospitalization, non-weight bearing and fasting) on AAs are still unclear. Further studies are warranted to examine this. Although AA profiles may have predicted reduced bone loss and lower risk of incident fracture (8), this cannot be tested in our case-control study as we collected blood samples after fracture occurrence and we cannot make causal inferences. Another limitation is that our study had a small sample size. However, based on our sample size calculation, the sample size is likely to be sufficient for testing the relationship between AAs and fracture. BMD of participants was not measured; protein and vitamin D intake levels were also not collected. However, a study suggested that the relationship between AAs and fracture risk was independent of diet and lifestyle factors (8). We did not have data on educational level and marital status in our study; these two factors are associated with sex and many conditions, including fractures (36–38). Data on body weight and height were self-reported. We cannot exclude the potential of confounding and information bias. Cases were identified from a hospital and controls were from a community-based population. Cases and controls were only matched on age and sex. Population selection and matching on limited factors may bias the reported results. Lastly, due to small sample size, subgroup results by fracture site and AA type lack reliability. Future validations are warranted.

## 5 Conclusion

In this matched case-control study, higher levels of AAs were negatively associated with osteoporotic fracture. This association appeared to be stronger for hip fracture than non-hip fracture. These findings, if confirmed by larger prospective studies, may have clinical implications for fracture healing. These findings extend our understandings about the beneficial effects of AAs on human health (i.e., muscle and kidney health) (14, 15).

## Data availability statement

The raw data supporting the conclusions of this article will be made available by the authors, without undue reservation.

## Ethics statement

The studies involving humans were approved by Institutional review boards (IRBs) of the School of Public Health, Jilin University. The studies were conducted in accordance with the local legislation and institutional requirements. The participants provided their written informed consent to participate in this study.

## Author contributions

BL: Conceptualization, Formal analysis, Methodology, Writing – review & editing. XinS: Conceptualization, Formal analysis, Methodology, Writing – review & editing. XW: Writing – original draft. CM: Writing – review & editing. WL: Writing – review & editing. LL: Writing – review & editing. XiaS: Writing – review & editing. BK: Funding acquisition, Writing – review & editing. SY: Conceptualization, Formal analysis, Methodology, Writing – original draft.
