# Supplementary material for: Association between amino acids and recent osteoporotic fracture: a matched incident case-control study
Source: Front Nutr. 2024 Mar 19;11:1360959. doi: 10.3389/fnut.2024.1360959 (PMC10985241; doi:10.3389/fnut.2024.1360959)
Supplement: Supplementary file 1 [file Data_Sheet_1.docx]

Supplementary Material

**Association between Amino Acids and Recent Osteoporotic Fracture: A Matched Case-control Study**

**Short title: Amino Acids and Fracture**

**Bing Liang^1^, Xinyan Shi^2^, Xinwei Wang^2^, Chao Ma^2^, William D. Leslie^3^, Lisa M. Lix^4^, Xianbao Shi^5^, Bo Kan^6^, Shuman Yang^1, 2, *^**

^1^Department of Endocrinology, The First Affiliated Hospital of Jinzhou Medical University, Jinzhou, Liaoning, China

^2^Department of Epidemiology and Biostatistics, School of Public Health, Jilin University, Changchun, Jilin, China

^3^Department of Internal Medicine, University of Manitoba, Winnipeg, Manitoba, Canada

^4^Department of Community Health Sciences, University of Manitoba, Winnipeg, Manitoba, Canada

^5^Department of Pharmacy, The First Affiliated Hospital of Jinzhou Medical University, Jinzhou, Liaoning, China

^6^ Department of Clinical Laboratory, The Second Hospital of Jilin University, Changchun, Jilin, China;

*** Correspondence:**

Dr. Shuman Yang

232-1163 Xinmin Street,

Department of Epidemiology and Biostatistics, Jilin University

Changchun, Jilin

China 130021

Phone: +86 18043635263

Fax: +86 043185645486

Email: [shumanyang@jlu.edu.cn](mailto:shumanyang@jlu.edu.cn)

ORCID: 0000-0002-9169-5850

**Supplementary Figure 1. Multivariable conditional logistic regression analysis of the association between amino acid levels and fracture adjusted for matching factors.**

**
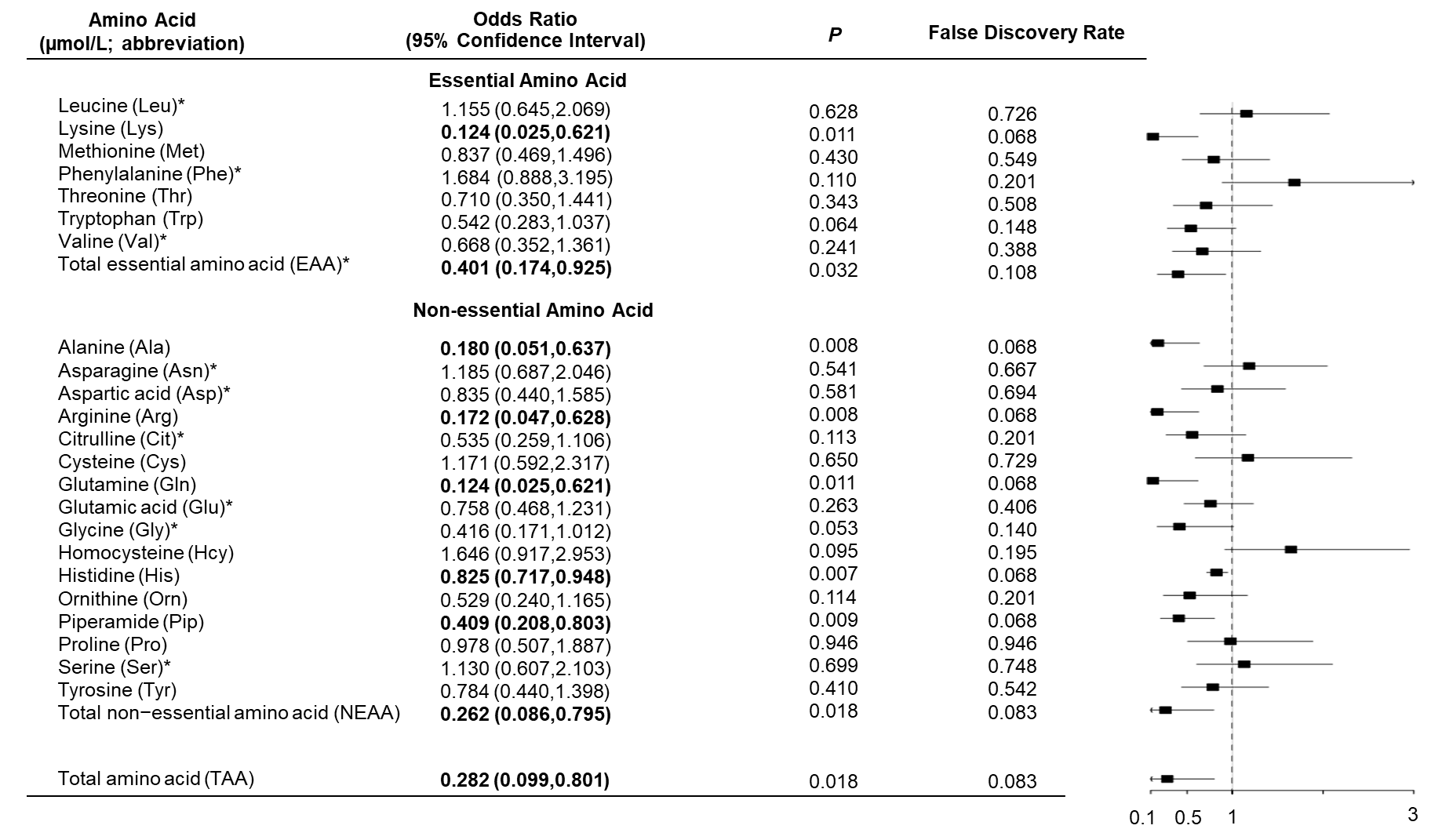
**

*Odds ratio presented per 1-SD increase on the logarithmic scale; all the other odds ratios are presented per 1-SD increase on the linear scale. Odds ratios were adjusted for age, sex, body mass index, physical activity, milk intake >1 time/week and falls.

Supplementary Figure 2. Multivariable logistic regression analysis of the association of aromatic amino acid and branched-chain amino acid levels with fracture.
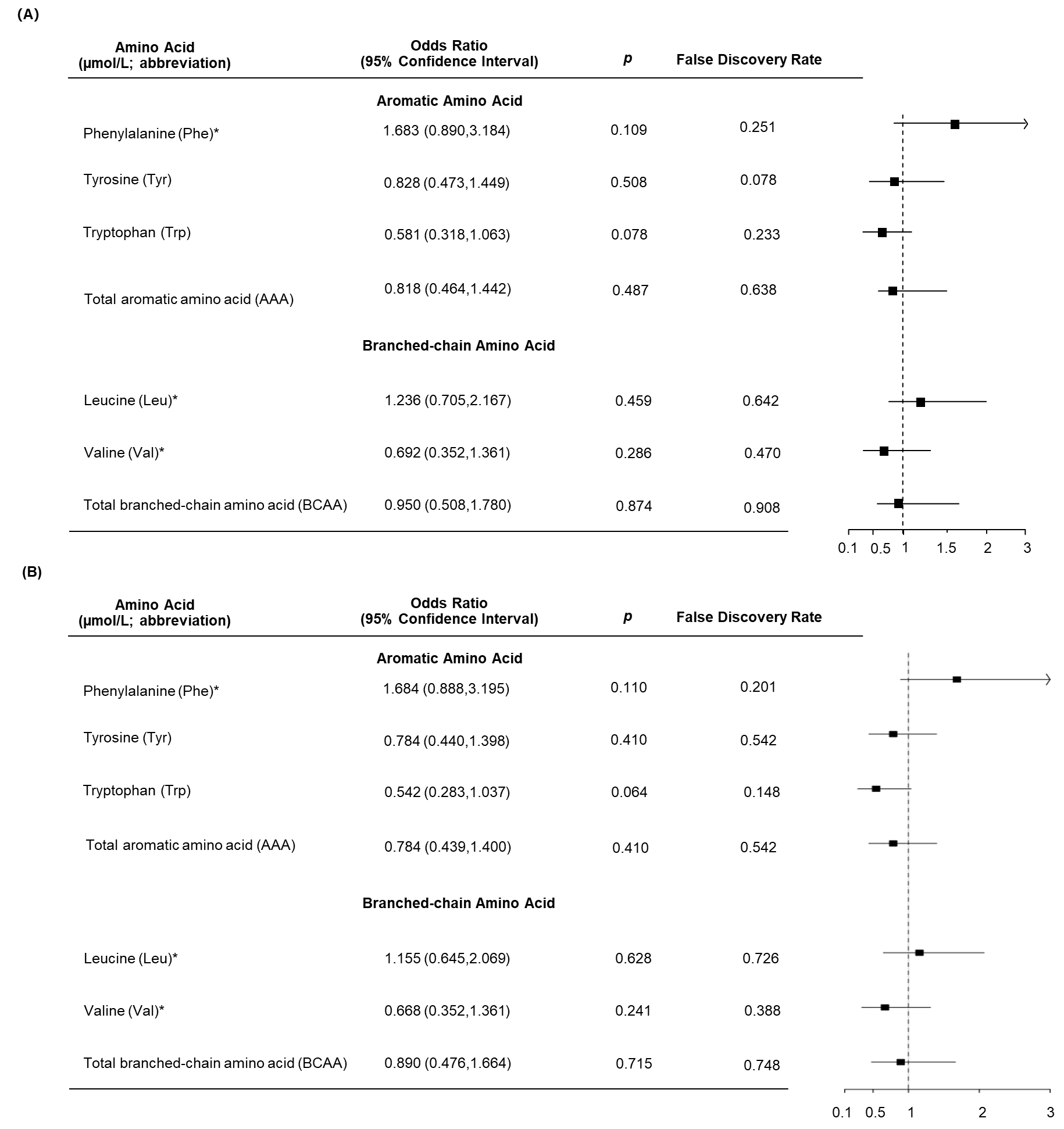


2

3

* Odds ratio presented per 1-SD increase on the logarithmic scale; all the other odds ratios are presented per 1-SD increase on the linear scale. (A) Odds ratios were adjusted for body mass index, physical activity, milk intake >1 time/week and falls; (B) Odds ratios were adjusted for age, sex, body mass index, physical activity, milk intake >1 time/week and falls.

**Supplementary Figure 3. Multivariable logistic regression analysis of the association between amino acid ratio and fracture adjusted for matching factors.**


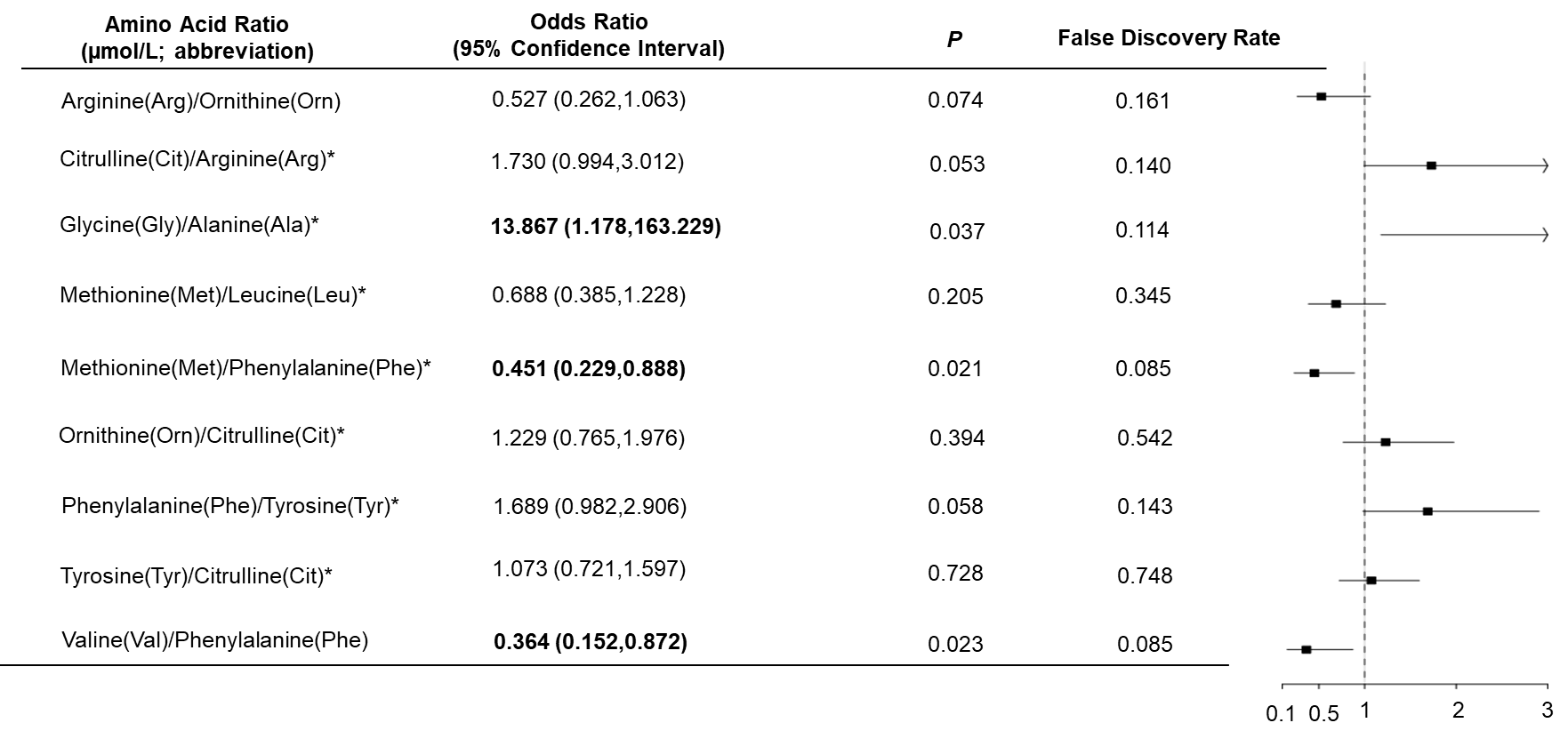


*Odds ratio presented per 1-SD increase on the logarithmic scale; all the other odds ratios are presented per 1-SD increase on the linear scale. Odds ratios were adjusted for age, sex, body mass index, physical activity, milk intake >1 time/week and falls.

**
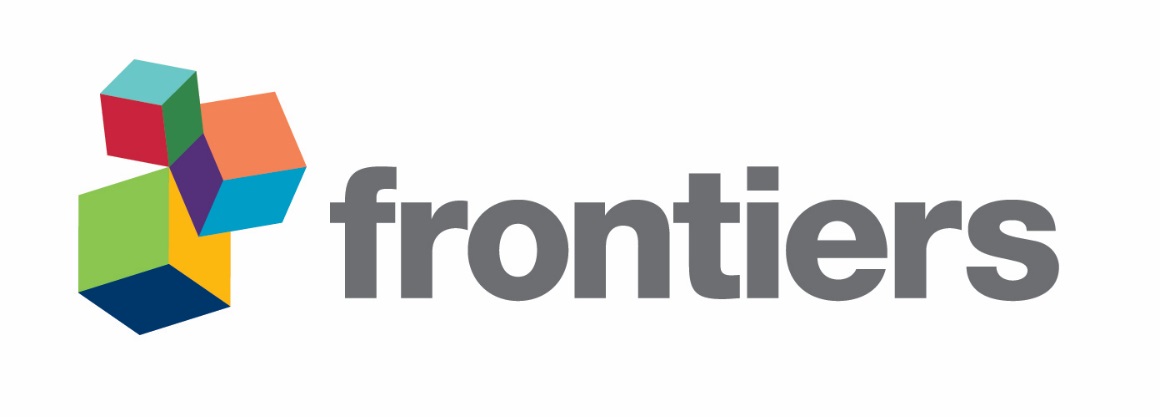
**
